# Shift from high-frequency to low-frequency episodic migraine in patients treated with Galcanezumab: results from two global randomized clinical trials

**DOI:** 10.1186/s10194-021-01222-w

**Published:** 2021-05-28

**Authors:** Jakub Jedynak, Eric Eross, Astrid Gendolla, Mallikarjuna Rettiganti, Virginia L. Stauffer

**Affiliations:** 1 Eli Lilly and Company, Indianapolis, IN 46285 USA; 2 Phoenix Headache Institute, Scottsdale, USA; 3 Praxis Gendolla, Essen, Germany

**Keywords:** QoL, MIDAS, MSQ-RFR, Episodic migraine, Sustained improvement, Migraine frequency

## Abstract

**Background:**

Patients with episodic migraine (EM) with a higher-frequency of migraine headache days (HFEM: 8–14 migraine headache days/month) have a greater disease burden and a higher risk of progressing to chronic migraine (CM) with associated acute treatment overuse versus those with low-frequency EM (LFEM: 4–7 migraine headache days/month). In this post hoc analysis, we assessed the proportions of patients who shifted from HFEM to LFEM and to very low-frequency EM (VLFEM: 0–3 migraine headache days/month) status following treatment with galcanezumab versus placebo.

**Methods:**

EVOLVE-1 and EVOLVE-2 were double-blind, Phase 3 studies in patients with EM. Patients (18–65 years) were randomized (2:1:1) to subcutaneous monthly injections of placebo, galcanezumab 120 mg (240 mg loading dose) or 240 mg, for up to 6 months. Data were pooled and endpoints were change from baseline in number of migraine headache days/month and patients who shifted from HFEM to LFEM or VLFEM status. Impact of change in HFEM status on migraine headache days/month, quality of life and disability was also assessed.

**Results:**

A total of 66% (1176/1773) patients from EVOLVE studies had HFEM status at baseline and were included in this analysis; placebo: 592, galcanezumab 120 mg: 294 and galcanezumab 240 mg: 290. At each month, both doses of galcanezumab resulted in a higher proportion of patients who shifted to 0–7 monthly headache days/month (VLFEM or LFEM status). Patients who shifted from HFEM at baseline to VLFEM status at Month 3, a relatively larger proportion of patients on galcanezumab 120 mg versus placebo remained at VLFEM status at Months 4–6; Months 4–5 for galcanezumab 240 mg versus placebo. Among the galcanezumab-treated patients who did-not-shift or shifted to LFEM or VLFEM status for ≥3 consecutive months until the end of the study, patients who shifted from HFEM to VLFEM status experienced the largest reduction in migraine headache days/month and the largest clinically meaningful improvements in daily functioning (MSQ-RFR) and disability (MIDAS).

**Conclusions:**

In patients with HFEM, treatment with galcanezumab (120 mg and 240 mg) significantly reduced migraine headache days/month, maintained remission status at subsequent months until the end of the study, and improved patients’ quality of life versus placebo.

**Trial registration:**

ClinicalTrials.gov Identifier: EVOLVE-1, NCT02614183; EVOLVE-2, NCT02614196.

**Supplementary Information:**

The online version contains supplementary material available at 10.1186/s10194-021-01222-w.

## Background

Migraine is a neurological disease characterized by high disability-adjusted life-years (DALYs) [1] and diminished health-related quality of life [2–4]. Patients with episodic migraine (EM) with a higher-frequency of migraine headache days, classified as high-frequency EM (HFEM: 8–14 migraine headache days/month) have a greater disease burden and a higher risk of progressing to chronic migraine (CM) with associated acute treatment overuse versus those with low-frequency EM (LFEM: 4–7 migraine headache days/month) [5, 6].

Galcanezumab is a humanized monoclonal antibody, that binds to calcitonin gene-related peptide (CGRP) and prevents its biological activity without blocking the CGRP receptor [7]. Galcanezumab 120 mg is approved globally as a once-monthly subcutaneous injection for the prevention of migraine. EVOLVE-1 and EVOLVE-2 were two identical Phase 3 studies in patients with EM who experienced an average of 9.1 migraine headache days/month at baseline [8–10]. During the 6 month double-blind treatment period, galcanezumab 120 mg or 240 mg resulted in a significantly larger reduction in migraine headache days/month. EVOLVE-1 showed a reduction of 4.7 days with galcanezumab 120 mg, 4.6 days with galcanezumab 240 mg versus 2.8 days with placebo [9]. The reduction in migraine headache days/month in EVOLVE-2 was 4.3 days with galcanezumab 120 mg, 4.2 days with galcanezumab 240 mg versus 2.3 days with placebo [9, 10]. Treatment with galcanezumab 120 mg and 240 mg also demonstrated statistically significant and clinically meaningful persistence of effect in patients with EM for ≥3 consecutive months and for 6 consecutive months [11]. A post hoc analysis of EVOLVE-1 and EVOLVE-2 studies demonstrated that approximately 40% of patients achieved 100% reduction in migraine headache days/month for at least 1 month of treatment and a relatively higher number of patients achieved 100% reduction in migraine headache days/month for at least 1 month in the last 3 months versus the first 3 months of the double-blind treatment period [8]. Galcanezumab 120 mg or 240 mg versus placebo demonstrated significant (*P* ≤ .001) reductions in migraine headache days/month together with improvement in Migraine-Specific Questionnaire Version 2.1 Role Function Restrictive domain (MSQv2.1 RFR), mean reduction in Migraine Disability Assessment (MIDAS) total score and ≥ 50% response rate in patients with LFEM or HFEM suggesting that galcanezumab is consistently effective in patients with EM, regardless of headache frequency [6]. In the current post hoc analysis from the EVOLVE-1 and EVOLVE-2 studies, we assessed the shift from HFEM status to LFEM status and, more importantly to very low-frequency EM (VLFEM: 0–3 migraine headache days/month) status following treatment with galcanezumab versus placebo. Additionally, changes in patient functioning and disability were assessed for patients shifting from HFEM status to LFEM status and from HFEM status to VLFEM status.

## Methods

Data for this post hoc analysis were from two identical Phase 3, multicenter, randomized, double-blind, placebo-controlled studies in patients with EM (EVOLVE-1, ClinicalTrials.gov identifier: NCT02614183; EVOLVE-2, ClinicalTrials.gov identifier: NCT02614196). Detailed descriptions of the study design have been previously published [9, 10]. Briefly, EVOLVE-1 and EVOLVE-2 studies consisted of an initial screening/washout period; a prospective lead-in period wherein the baseline frequencies of migraine headache days were determined; a 6 month double-blind treatment period; and a 4 month posttreatment washout period. During the 6 month double-blind treatment period, patients were randomized 2:1:1 to receive either placebo, subcutaneous injections of galcanezumab 120 mg (following a loading dose of galcanezumab 240 mg) or galcanezumab 240 mg on a monthly basis. With the exception of medications containing opioids or barbiturates, which were allowed up to 3 days a month, acute migraine medications use was permitted during the baseline and treatment period. Patients were included if they were aged between 18 to 65 years with a diagnosis of migraine with or without aura as defined by ICHD-3β criteria [12], had a diagnosis of migraine at least 1 year before enrollment, 4 to 14 migraine headache days/month, at least 2-migraine attacks within the past 3 months prior to study entry.

### Study assessments

There were four key objectives in this post hoc analysis. We aimed to determine and compare the following between the galcanezumab and placebo groups:

1. the proportion of patients who shifted from HFEM status at baseline to LFEM status or from HFEM status at baseline to VLFEM status at each individual month,
2. the proportion of patients who maintained in VLFEM status during Months 4–6 among those who shifted to VLFEM at Month 3,
3. the proportion of patients who shifted from HFEM status to VLFEM status anytime during the double-blind treatment period and who maintained the shift for ≥3 consecutive months until the end of the study and
4. the change from baseline to Month 6 in monthly migraine headache days, MSQv2.1 RFR domain (patient functioning) and MIDAS total score (patient disability) among patients who either shifted from HFEM status to LFEM status for ≥3 consecutive months and until the end of the study, or from HFEM status to VLFEM status for ≥3 consecutive months and until the end of the study, or those who did not adequately shift during the treatment phase.

Migraine headache frequency groups were defined using the following notations/ranges: HFEM = 8–14 migraine headache days/month, calculated as ≥8 to ≤14; LFEM = 4–7 migraine headache days/month, calculated as ≥4 to < 8 and VLFEM = 0–3 migraine headache days/month, calculated as 0 to < 4. At baseline, the HFEM status group was identified by the previously mentioned range (≥8 to ≤14 migraine headache days/month). However, during the double-blind treatment phase, patients could have experienced more than 14 migraine headache days/month and therefore the high frequency group was defined as ≥8 migraine headache days/month.

The patient’s functioning following treatment with galcanezumab was measured using MSQv2.1 RFR domain change score [13, 14]. The MSQv2.1 is a self-administered questionnaire that assesses the effect of migraine on work or daily activities, relationships with family and friends, leisure time, productivity, concentration, energy, tiredness and feelings over the past 4 weeks [13, 14]. Specifically, the RFR measures the degree to which migraine limits the performance of usual activities. The participants rated the items on a standard 6-point ordered-categorical scale, ranging from “none of the time” to “all of the time” [13] . The RFR raw scores were transformed to ranges of 0–100, with 100 indicating the best functional health status and a positive change in scores reflecting functional improvement [5]. Headache-related disability associated with missed or reduced productivity at work or home and social events was assessed using the MIDAS, a 5-item questionnaire that quantifies disability over a 3 month period. Participants can rate from “little or no disability” (grade I, score 0 to 5) to “severe disability” (grade IV, score 21 or above) and overall scores range from 0 to 270 with higher scores indicating greater disability) [15]. Both the MSQv2.1 [14] and MIDAS [16, 17] instruments are considered valid and highly reliable and correlates well with clinical impression.

### Statistical analysis

For the purpose of this analysis, patients were categorized into the following three categories based on their frequency of migraine headache days observed during their prospective baseline period: HFEM (8–14 migraine headache days/months), LFEM (4–7 migraine headache days/month) and VLFEM status (0–3 migraine headache days/month). Analyses were performed in the intention-to-treat HFEM population, which included patients who had been randomly assigned to a study treatment and received at least one dose of either placebo or galcanezumab.

Among patients categorized as HFEM status at baseline, the proportion of patients who shifted to LFEM status during the treatment period were estimated with a generalized linear mixed repeated measures model (GLIMMIX) for binary outcomes with a logit link function. This model included the baseline migraine headache days as a covariate and the following fixed effects: treatment, month, study, treatment-by-month interaction, baseline by month interaction and pooled region. A similar model was used to analyze the shift from HFEM status at baseline to VLFEM status at each month during the treatment period. Among the patients who shifted from HFEM status to VLFEM status at Month 3, the proportion of patients in each treatment group who remained at VLFEM in subsequent months was estimated using a GLIMMIX model after including treatment group, baseline migraine headache days, treatment by month interaction effect and the study indicator. In all repeated measures models, correlation among repeated observations on the same patient was accounted for assuming a variance components correlation structure.

The proportion of patients who shifted from HFEM status at baseline to VLFEM status anytime during the double-blind treatment period and who maintained that shift for ≥3consecutive months and until the end of the treatment period was analyzed using a logistic regression model that included the baseline migraine headache days, treatment indicator, pooled region and study indicator.

To assess whether the shift in migraine frequencies from HFEM status to LFEM or HFEM to VLFEM status was associated with corresponding improvements in functional or disability scores at the end of the double-blind period, the changes from baseline to 6 months in MSQv2.1 RFR domain score and MIDAS total score were estimated and compared among the following three groups of patients: those who shifted from HFEM status to LFEM status for at least three consecutive months and until the end of the study, those who shifted from HFEM status to VLFEM status for at least three consecutive months and until the end of the study and those who did not sufficiently shift. After adjusting for baseline, these shifts were determined using an analysis of covariance model.

#### Handling of missing data

In computing the number of migraine headache days/month, if for a particular month, the number of days with non-missing data in the daily diary did not equal to 30 days, the number of migraine headache days was normalized to a 30-day period by multiplying the number of observed migraine headache days by the number of non-missing diary days and multiplying by 30. For example, if a patient reported seven migraine headache days/month on 27/30 registered entries in their diary, the 7 migraine headache days total would be adjusted to (7/27)*30 = 7.78 migraine headache days/month and the patient would be labeled as LFEM. If the compliance rate for a monthly interval was ≤50%, then the number of migraine headache days for that month was assumed to be missing. All analyses included patients in the intent-to-treat population who had non-missing value at baseline and at least one valid outcome observed during the double-blind phase. No other adjustments such as multiple imputation was done to handle missing data.

#### General considerations

Details on patient enrollment, sample size calculation, randomization and blinding for the primary studies were previously published [9, 10]. Data were summarized using summary statistics such as mean and standard deviation for numeric variables and frequency and percentage for categorical variables. Least square (LS) means were estimated using the models’ assumed average values of covariates and may be different from the observed data shown. Comparisons between groups were presented using odds ratios (OR) and 95% confidence intervals (CI) for GLIMMIX models or logistic regression models and LS mean differences and 95% confidence intervals for analysis of covariance models. All analyses were done using SAS Enterprise Guide Version 7.1 for Windows. All statistical tests were two-sided assuming a significance level of 5%.

## Results

### Patient disposition and demographics

Of the 1773 patients with EM from the EVOLVE-1 and EVOLVE-2 studies (placebo: 894, galcanezumab 120 mg: 444, galcanezumab 240 mg, 435), 66% of patients (1176/1773) had HFEM status at baseline and were included in this post hoc analysis. Of these, 592 patients received placebo, 294 received galcanezumab 120 mg and 290 received galcanezumab 240 mg at baseline (Table 1).

**Table 1:** Baseline characteristics in patients with HFEM status

	Placebo (***n*** = 592)	GMB 120 mg (***n*** = 294)	GMB 240 mg (***n*** = 290)
Age, years, mean (SD)	41.5 (11.3)	40.6 (11.4)	40.1 (11.5)
Female, n (%)	518 (87.5)	249 (84.7)	251 (86.6)
Years since migraine diagnosis, mean (SD)	20.3 (12.3)	20.7 (12.1)	19.5 (11.9)
Number of migraine headache days, mean (SD)	10.9 (2.0)	10.9 (2.0)	10.7 (2.0)
MSQ v2.1 Role Function Restrictive (SD)	49.9 (15.0) ^#^	49.5 (15.2)	48.8 (16.7)^##^
MIDAS total score, mean (SD)	36.9 (31.5) ^#^	35.3 (30.2)	37.2 (29.7)^##^

^**#**^***n*** **= 586;** ^**##**^***n*** **= 286**

**Abbreviations:** *GMB* galcanezumab, *HFEM* high-frequency episodic migraine, *MIDAS* Migraine Disability Assessment, *MSQv2.1 RFR* Migraine-Specific Questionnaire Version 2.1 Role Function Restrictive; SD, standard deviation

### Monthly shift from HFEM status to LFEM or from HFEM status to VLFEM status

**Fig. 1:**
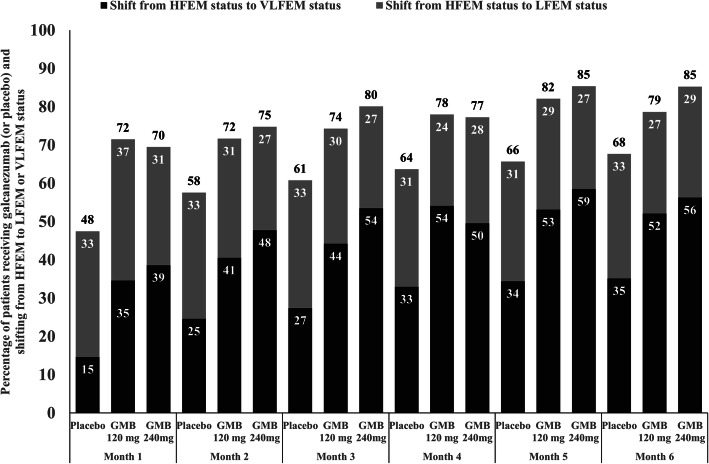
Percentage of HFEM patients at baseline receiving galcanezumab (versus placebo) and shifting from HFEM status to LFEM status, or HFEM to VLFEM status at each month during the treatment period. Abbreviations: GMB, galcanezumab; HFEM, high-frequency episodic migraine; LFEM, low-frequency episodic migraine; n, number of patients who were categorized as HFEM at baseline with a baseline value and at least one post-baseline value recorded; VLFEM, very low-frequency episodic migraine. Note: The percentages shown in the figure are raw values at each month and treatment group. *P* values comparing galcanezumab vs placebo are from the following GLIMMIX model for repeated measures: Shift from HFEM to VLFEM indicator = baseline migraine headache days/month, treatment group, month, treatment x month interaction, and study indicator (EVOLVE-1 and EVOLVE-2)

At each month, both doses of galcanezumab (120 or 240 mg) resulted in a higher proportion of patients who shifted to 0–7 monthly headache days/month (VLFEM or LFEM status). This treatment difference was primarily driven by a greater proportion of patients who shifted from HFEM to VLFEM status in galcanezumab versus placebo treated patients (Fig. 1). Numerically, a greater proportion of patients shifted from HFEM to LFEM status (4–7 migraine headache days/month) in the placebo compared to galcanezumab group. However, this treatment difference is mostly due to a significantly greater proportion of galcanezumab patients shifting to VLFEM status compared with placebo. It is not clear from the static figure whether a patient who shifted to LFEM or VLFEM at Month 1 remained in the same frequency status group in subsequent months. To further examine if there was substantial patient movement over time between frequency categories, we created a data animation to examine the shift of galcanezumab- and placebo-treated individual patients from HFEM status to LFEM or VLFEM status across each month (Additional file 1: Video Fig. S1). In summary, the monthly percentage of patients who achieved LFEM status appeared constant due to patients shifting from either LFEM to VLFEM status or LFEM to HFEM status across Months 2–6.

### Proportion of patients shifting from HFEM status to VLFEM status at month 3 and maintaining VLFEM status during months 4–6

**Fig. 2:**
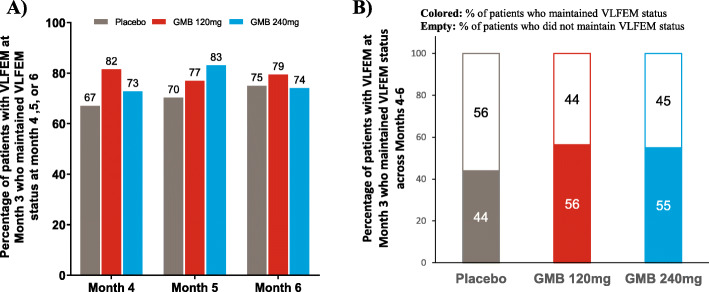
Percentage of patients who achieve VLFEM at Month 3 and (**a**) maintained VLFEM status at each of Months 4, 5 or 6, (**b**) maintained VLFEM status across Months 4, 5 or 6. Abbreviations: CI, confidence interval; GMB, galcanezumab; HFEM, high-frequency episodic migraine; n, number of patients who were categorized as HFEM at baseline with a baseline value and at least one post-baseline value recorded; OR, odds ratio; VLFEM, very low-frequency episodic migraine

In patients who shifted from HFEM status at baseline to VLFEM status at Month 3, a relatively larger proportion of patients on galcanezumab 120 mg remained at VLFEM status at Months 4, 5, or 6 compared with placebo. A greater proportion of patients receiving galcanezumab 240 mg remained at VLFEM status at Month 4 or 5 compared with placebo (Fig. 2). These results for patients administered galcanezumab (120 and 240 mg) were mirrored in the accompanying data animation (Additional file 2: Video Fig. S2). In patients who maintained their VLFEM status from Month 3 until the end of the study, 56% and 55% of patients received galcanezumab 120 mg or 240 mg versus 44% who received placebo.

### Proportion of patients shifting from HFEM status to VLFEM status anytime during the double-blind treatment period and maintaining the response for ≥3 consecutive months until the end of the study

**Fig. 3:**
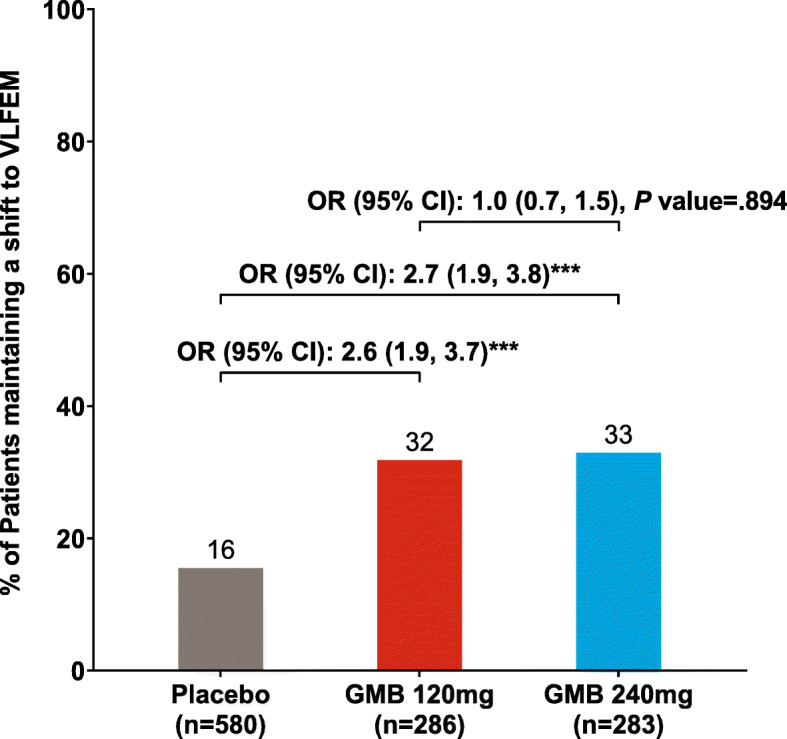
Percentage of patients shifting from HFEM to VLFEM status anytime during the double-blind treatment period and maintaining their shift from HFEM status to VLFEM status for ≥ 3 consecutive months until the end of the study (patients with HFEM at baseline). ***P < .001 versus placebo from logistic regression analysis. Abbreviations: CI, confidence interval; GMB, galcanezumab; HFEM, high-frequency episodic migraine; n, number of patients who were categorized as HFEM at baseline with a baseline value and at least one post-baseline value recorded; OR, odds ratio; VLFEM, very low-frequency episodic migraine

Both doses of galcanezumab 120 mg or 240 mg compared to placebo resulted in a significantly (*P* < .001) larger proportion of patients who shifted from HFEM status to VLFEM status for ≥3 consecutive months and until the end of the study (Fig. 3). The odds of maintaining a shift from HFEM status to VLFEM status until the end of the study was 2.6 times greater for the galcanezumab 120 mg group compared with placebo (95% CI, *P* value: 1.9 to 3.7, ≤.001). The corresponding odds ratio for galcanezumab 240 mg versus placebo was 2.7 (95% CI 1.9 to 3.8, *P* ≤ .001). No significant difference was observed between the two galcanezumab doses (OR [95% CI], *P* value: 1.0 [0.7, 1.5], *P* = .894).

### Impact of change in HFEM status on migraine headache days/month, quality of life and disability

**Fig. 4:**
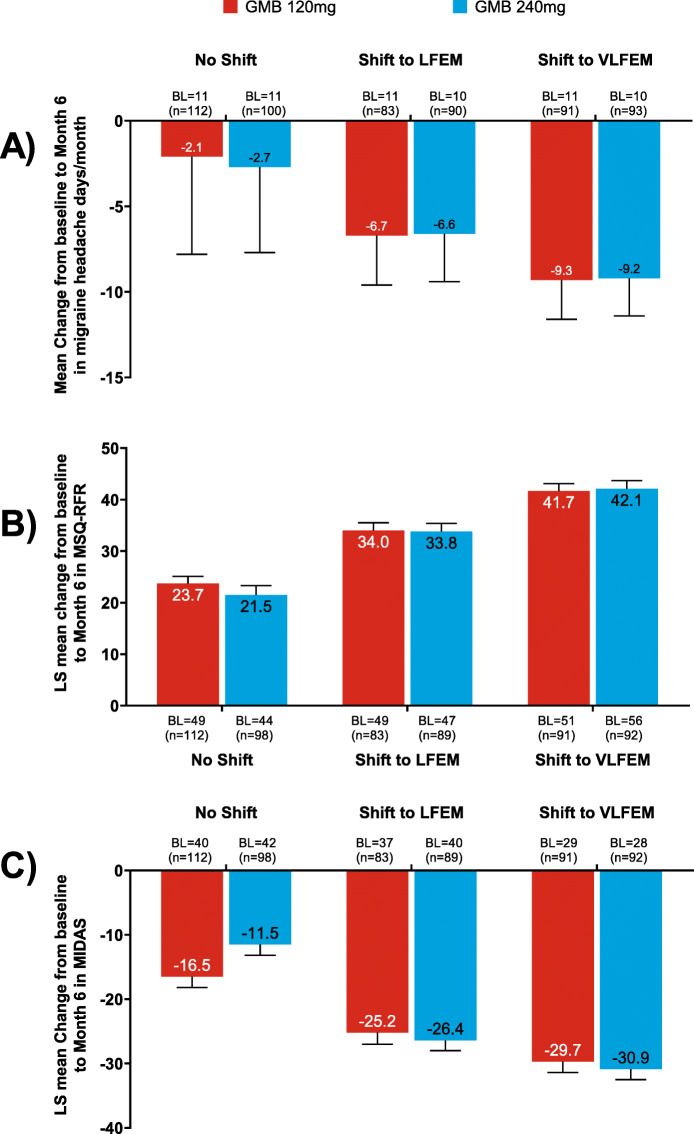
Change from baseline to Month 6 in HFEM patients receiving galcanezumab who 1) did not shift, 2) shifted to LFEM or 3) VLFEM for ≥ 3 consecutive months until the end of the study for (**a**) migraine headache days/month, (**b**) MSQ-RFR score and (**c**) MIDAS total score. All *P* < .001 shift from ‘HFEM status to LFEM status’ versus ‘no shift’ and to ‘VLFEM status’ versus ‘no shift’. The between group comparisons for migraine headache days/month (panel **a**) were done using ANOVA with raw mean changes and standard deviation shown, whereas MSQ (panel **b**) and MIDAS (panel **c**) were analyzed using ANCOVA models adjusted for covariates with estimated least squares means and standard errors displayed. Abbreviations: ANOVA, Analysis of Variance; ANCOVA, Analysis of Covariance; BL, baseline; GMB, galcanezumab; HFEM, high-frequency episodic migraine; HFEM, low-frequency episodic migraine; MIDAS, Migraine Disability Index, MSQv2.1 RFR, Migraine-Specific Questionnaire Version 2.1 Role Function Restrictive, VLFEM, very low-frequency episodic migraine

For HFEM galcanezumab-treated patients who did not shift or shifted to LFEM or VLFEM for ≥3 consecutive months until the end of the study, a greater reduction in the mean change from baseline to Month 6 in migraine headache days/month was observed with increasing shift magnitude from HFEM status (Fig. 4a). The LS mean change (SD) from baseline with galcanezumab 120 mg/240 mg in migraine headache days/month was − 6.7 (2.9) and − 6.6 (2.8) in patients who shifted to LFEM status, − 9.3 (2.3) and − 9.2 (2.2) in patients who shifted to VLFEM status. All of these changes were significantly lower compared with a LS mean change of − 2.1 (5.7) and − 2.7 (5.0) with galcanezumab 120 mg or 240 mg for patients who did not adequately shift (*P* value <.001 for both comparisons).

For HFEM galcanezumab-treated patients that did not shift or shifted to LFEM or VLFEM for ≥3 consecutive months until the end of the study, the LS mean change from baseline to Month 6 in MSQv2.1 RFR was progressively higher with increasing magnitude of shift from HFEM status (indicating improvement; Fig. 4b). The LS mean change (SE) from baseline to Month 6 in MSQv2.1 RFR with galcanezumab 120 mg/240 mg was 34.0 (1.5) and 33.8 (1.6) in patients who shifted to LFEM status, 41.7 (1.4) and 42.1 (1.6) in patients who shifted to VLFEM status. These shifts were significantly larger compared with a LS mean (SE) change of 23.7 (1.4) and 21.5 (1.8) with galcanezumab 120 mg/240 mg for patients who did not adequately shift (*P* < .001 for both comparisons, Fig. 4).

For HFEM galcanezumab-treated patients, the LS mean change from baseline to Month 6 in MIDAS increased in magnitude from patients who did not shift to patients who shifted to LFEM or VLFEM for ≥3 consecutive months until the end of the study. The LS mean change (SE) from baseline with galcanezumab 120 mg/240 mg in MIDAS was − 25.2 (1.8) and − 26.4 (1.6) in patients who shifted to LFEM status, − 29.7 (1.7) and − 30.9 (1.6) in patients who shifted to VLFEM status, which were both significantly lower compared with a LS mean change of − 16.5 (1.7) and − 11.5 (1.7) with galcanezumab 120 mg or 240 mg for patients who did not adequately shift (*P* value <.001 for both comparisons; Fig. 4c).

## Discussion

Migraine is a highly debilitating disease in both its episodic and chronic forms, with CM imposing more substantial individual and socioeconomic burden as described by various population-based studies [18–20]. Reductions in monthly migraine headache days and patient functioning have been reported with galcanezumab in patients with CM or EM [9, 10]. Here, we report results from a post hoc analysis of the galcanezumab EVOLVE studies in a subgroup of patients with baseline HFEM status. This analysis evaluated the proportion of patients who shifted from HFEM status to LFEM or VLFEM status during the double-blind treatment period. The analysis also evaluated the proportion of patients who shifted to VLFEM status for ≥3 consecutive months either at Month 3 or at any time during the double-blind treatment period and maintained that shift until the end of the double-blind treatment period. The current findings are of significance as the identified population has a greater disease burden and is at higher risk of progression from EM to CM [6, 21–23].

Both doses of galcanezumab and placebo led to an increase in the proportion of patients who shifted from HFEM status to LFEM or VLFEM status. At the end of Month 1, 34.6% of patients on galcanezumab 120 mg and 38.7% on galcanezumab 240 mg experienced a larger shift from HFEM status to VLFEM status compared to 14.7% on placebo. This shift in galcanezumab-treated patients was sustained versus placebo at each month thereafter until Month 6 wherein 52.2% of patients on galcanezumab 120 mg and 56.3% on galcanezumab 240 mg achieved VLFEM status versus 35.2% on placebo. In general, a relatively greater proportion of galcanezumab-treated patients shifted from HFEM status to VLFEM status than HFEM to LFEM status each month. Interestingly, the percentage of patients who achieved LFEM status did not differ over time or between treatment groups. To further investigate this result, we examined individual patient-level status shifts through data animations (Additional file 1: Video Fig. S1) which showed that patients with LFEM status either maintained their LFEM status, reverted back to HFEM status, or shifted to VLFEM status the following month. Correspondingly, patients losing LFEM status were replaced by patients with HFEM and VLFEM status from the previous month. These complex monthly dynamics can be difficult to represent in static mediums and can be better understood using individual patient-level transitions. Overall, LFEM status appears to be temporary with the majority of galcanezumab patients compared to placebo shifting towards VLFEM status.

We next examined the sustainability of VLFEM status by examining subsequent shifts in patients who achieved VLFEM status at Month 3. We observed a large percentage of galcanezumab-treated patients who subsequently maintained VLFEM status at either Months 4, 5, or 6. This was also demonstrated in the accompanying data animation (Additional file 2: Video Fig. S2) as approximately 80% of patients on galcanezumab achieved VLFEM status at Months 4, 5, or 6. However, the treatment differences versus placebo were not very robust. By selecting galcanezumab- and placebo-treated patients who achieved VLFEM status at Month 3, we identified “early” responders who, not surprisingly, maintained their response across the remaining months in the study. However, when we examined patients who sustained VLFEM status across consecutive months, a total of 56.4% patients on galcanezumab 120 mg and 55.0% patients on galcanezumab 240 mg versus 43.9% on placebo maintained their VLFEM status across Months 4, 5 and 6. Examining continuous VLFEM status is important as sustained efficacy would only be achieved by patients who maintain their VLFEM status across consecutive months. These findings are also consistent with those reported earlier wherein patients who responded to treatment at Month 3 were more likely to maintain their response status for a longer duration of time [24].

To further examine the sustainability of VLFEM status in our study, we measured the percentage of patients who shifted to VLFEM status for three or more months and maintained VLFEM status until the end of the study [11]. In this more rigorous analysis, the estimated proportions of patients shifting from HFEM to VLFEM status at any time during the double-blind period and maintaining that shift for ≥3 consecutive months until the end of study was significantly larger for galcanezumab 120 mg (31.8%) or galcanezumab 240 mg (32.9%) versus placebo (15.5%). These results suggest that the effects of galcanezumab versus placebo are likely to be long term and sustainable.

Among the galcanezumab-treated patients who did not shift or shifted to LFEM or VLFEM status for ≥3 consecutive months until the end of the study, patients who shifted from HFEM status to VLFEM status experienced the largest reduction in migraine headache days/month and the largest clinically meaningful improvements in daily functioning (per MSQ-RFR) and disability (MIDAS). At baseline, HFEM patients reported MSQ-RFR domain scores of less than 50%, which indicated considerable functional impairment due to the degree which migraine limits the performance of usual activities over the last 4 weeks. Following 6 months of galcanezumab treatment, the MSQ-RFR domain increased from baseline in the HFEM to LFEM group to over 80% of the total possible score for both galcanezumab dose groups and in the HFEM to VLFEM group to over 90% for both galcanezumab dose groups compared to approximately 70% for both galcanezumab dose groups who did not shift to LFEM or VLFEM status. These results indicate substantial increases in functional capabilities related to relationships with friends and family, leisure time activities, work or daily activities, productivity, concentration, energy and tiredness. Baseline MIDAS total score also demonstrated that HFEM patients were severely disabled due to migraine. The magnitude of the MIDAS total score reduction was lower in HFEM patients who did not shift compared to those who shifted to LFEM or VLFEM status. Correspondingly, categorical grade-level changes were observed in patients who shifted from HFEM (severe disability) to LFEM (moderate disability) or VLFEM (little to no disability) whereas patients who did not shift remained severely disabled. These results indicate substantial reductions in disabilities related to everyday home, work/school and social/leisure activities. Overall, these current findings demonstrated that treatment with galcanezumab 120 mg or 240 mg significantly reduces the number of migraine headache days/month together with an improvement in day-to-day functioning and reduction in headache disability.

### Limitation

This post hoc analysis was not pre-specified per the study design of EVOLVE-1 and EVOLVE-2, which limits the ability to make definitive conclusions. Additionally, per the inclusion criteria for the EVOLVE studies, a migraine headache day was defined as both the presence of migraine headache and probable migraine headache [9, 10]. This definition could make direct comparisons to migraine headache and, exclusively, to probable migraine difficult.

## Conclusions

In conclusion, both doses of galcanezumab significantly increased the percentage of HFEM patients shifting to VLFEM status compared to placebo. Importantly, a significantly greater percentage of galcanezumab treated patients compared to placebo shifted from HFEM to VLFEM at any time during the double-blind treatment period and subsequently maintained that status for 3 or more consecutive months until the end of the study. The sustained shift from HFEM to VLFEM status was accompanied by clinically meaningful improvements in quality of life measures such as MSQ-RFR and MIDAS total scores at Month 6. Overall, these novel findings in HFEM patients, a migraine population that possesses greater disease burden and higher risk for progression from EM to CM, demonstrated that galcanezumab treatment produces a sustained reduction in migraine frequency that is accompanied by robust and clinically meaningful improvements in quality of life.

## Supplementary Information

**Additional file 1: Video Fig. S1.** Individual patients with HFEM status receiving galcanezumab (versus placebo) and shifting from HFEM status to LFEM status or VLFEM status at each month during the treatment period. For the data animation, both doses of galcanezumab (120 mg and 240 mg) were pooled to form a galcanezumab only group where each dot represents an individual patient in the pooled EVOLVE-1 and EVOLVE-2 studies. At month 1, only the HFEM population is visible in the placebo (left) and galcanezumab (right) treatment arms. The colors in each treatment group reflect migraine headache days/month frequency as follows: HFEM (≥8 and ≤ 14 migraine headache days/months) = yellow; LFEM (≥4 and < 8 migraine headache days/months) = olive; and VLFEM (≤3 migraine headache days/months) = green. Patients who transition from one frequency status to another are highlighted with a black outline.

**Additional file 2: Video Fig. S2.** Individual patients with VLFEM status at Month 3 and their individual response to galcanezumab 120 or 240 mg during Months 4, 5, or 6. For the data animation, both doses of galcanezumab (120 mg and 240 mg) were pooled to form a galcanezumab only group wherein each dot represents an individual patient in the trial. At month 1, only the HFEM population is visible in the placebo (left) and galcanezumab (right) treatment arms. The colors in each treatment group reflect migraine headache days/month frequency as follows: HFEM (8–14 migraine headache days/months) = yellow; LFEM (4–7 migraine headache days/months) = olive; and VLFEM (0–3 migraine headache days/months) = green. Patients who transition from one frequency status to another are highlighted with a black outline. At month 3, only patients who achieved VLFEM status with galcanezumab and placebo treatment are visible as other groups have been removed. Subsequent animations display their frequency status over Months 4–6.

## Data Availability

Individual participant data collected during the trial, after anonymization, with the exception of pharmacokinetic or genetic data. Data are available to request 6 months after the indication studied has been approved in the US and EU and after primary publication acceptance, whichever is later. No expiration date of data requests is currently set once data are made available. Access is provided after a proposal has been approved by an independent review committee identified for this purpose and after receipt of a signed data sharing agreement. Data and documents, including the study protocol, statistical analysis plan, clinical study report, blank or annotated case report forms, will be provided in a secure data sharing environment. For details on submitting a request, see the instructions provided at **www.vivli.org.**

## Abbreviations

CGRP: Calcitonin gene-related peptide
CI: Confidence interval
CM: Chronic migraine
EM: Episodic migraine
HFEM: High-frequency episodic migraine (8–14 migraine headache days/month, calculated as ≥8 to ≤14)
LFEM: Low-frequency episodic migraine (4–7 migraine headache days/month, calculated as ≥4 to < 8)
LS: Least square
MIDAS: Migraine Disability Assessment
MSQv2.1: Migraine-Specific Questionnaire Version 2.1
OR: Odds ratio
RFR: Role Function Restrictive
SD: Standard deviation
SE: Standard error
VLFEM: Very low-frequency episodic migraine (0–3 migraine headache days/month, calculated as 0 to < 4)
